# Identification of neural connectivity signatures of autism using machine learning

**DOI:** 10.3389/fnhum.2013.00670

**Published:** 2013-10-17

**Authors:** Gopikrishna Deshpande, Lauren E. Libero, Karthik R. Sreenivasan, Hrishikesh D. Deshpande, Rajesh K. Kana

**Affiliations:** ^1^AU MRI Research Center, Department of Electrical and Computer Engineering, Auburn UniversityAuburn, AL, USA; ^2^Department of Psychology, Auburn UniversityAuburn, AL, USA; ^3^Department of Psychology, University of Alabama at BirminghamBirmingham, AL, USA

**Keywords:** autism, effective connectivity, fMRI, classification, machine learning, theory-of-mind

## Abstract

Alterations in interregional neural connectivity have been suggested as a signature of the pathobiology of autism. There have been many reports of functional and anatomical connectivity being altered while individuals with autism are engaged in complex cognitive and social tasks. Although disrupted instantaneous correlation between cortical regions observed from functional MRI is considered to be an explanatory model for autism, the causal influence of a brain area on another (effective connectivity) is a vital link missing in these studies. The current study focuses on addressing this in an fMRI study of Theory-of-Mind (ToM) in 15 high-functioning adolescents and adults with autism and 15 typically developing control participants. Participants viewed a series of comic strip vignettes in the MRI scanner and were asked to choose the most logical end to the story from three alternatives, separately for trials involving physical and intentional causality. The mean time series, extracted from 18 activated regions of interest, were processed using a multivariate autoregressive model (MVAR) to obtain the causality matrices for each of the 30 participants. These causal connectivity weights, along with assessment scores, functional connectivity values, and fractional anisotropy obtained from DTI data for each participant, were submitted to a recursive cluster elimination based support vector machine classifier to determine the accuracy with which the classifier can predict a novel participant's group membership (autism or control). We found a maximum classification accuracy of 95.9% with 19 features which had the highest discriminative ability between the groups. All of the 19 features were effective connectivity paths, indicating that causal information may be critical in discriminating between autism and control groups. These effective connectivity paths were also found to be significantly greater in controls as compared to ASD participants and consisted predominantly of outputs from the fusiform face area and middle temporal gyrus indicating impaired connectivity in ASD participants, particularly in the social brain areas. These findings collectively point toward the fact that alterations in causal connectivity in the brain in ASD could serve as a potential non-invasive neuroimaging signature for autism.

## Introduction

A biological origin for autism spectrum disorders (ASD) had been proposed even in the earliest published accounts of the disorder (Kanner, 1943; Asperger, 1944). Despite several decades of research since then, a focal neurobiological marker for autism has been rather elusive. Brain imaging techniques in the last decade, particularly functional and structural MRI, have pointed to disrupted cortical connectivity as a defining neural feature of ASD (Kana et al., 2011; Just et al., 2012). Neuroimaging studies have reported functional under connectivity (weaker synchronization of activated brain areas) between frontal and posterior brain areas (Just et al., 2004, 2007; Villalobos et al., 2005; Kana et al., 2006, 2007, 2009; Koshino et al., 2008; Mason et al., 2008; Solomon et al., 2009; Damarla et al., 2010; Jones et al., 2010; Mizuno et al., 2011; Schipul et al., 2011), and intact or increased functional connectivity within relatively posterior brain areas (Villalobos et al., 2005; Kana et al., 2006; Damarla et al., 2010; Kana et al., under review). Similar findings have also been reported during task-free resting state in autism (Cherkassky et al., 2006; Assaf et al., 2010; Murdaugh et al., 2012). Furthermore, diffusion tensor imaging (DTI) studies have reported disruptions in anatomical connectivity in ASD (Barnea-Goraly et al., 2004, 2010; Alexander et al., 2007; Keller et al., 2007; Jou et al., 2011; see Travers et al., 2012 for a review). Although there is converging evidence for connection abnormalities, the neural connectivity model of ASD is based primarily on functional connectivity, with some contributing evidence from white matter integrity. While the insights gained from these models are valuable, functional connectivity is a method for assessing zero-lag correlations, and does not provide insight into the time-lagged relationships and direction of such causal influence.

Effective connectivity, on the other hand, refers to the influence one neural system exerts over another with respect to a given experimental context (Buchel and Friston, 2000), thus helping uncover more information about how brain areas communicate. Effective connectivity can provide information about the transfer of information from one node to another, and differentiate between top-down vs. bottom-up effects. Thus, effective connectivity findings have enriched models of cognitive function by emphasizing the dynamic and interactive nature of neural instantiations (McIntosh et al., 2010). Studying such interactions is important not only for understanding typical brain functioning, but also is critical in learning more about diseases. Considering relatively consistent reports of disruptions in functional connectivity in ASD, it is perhaps a logical and valuable next step to study how information transfer is accomplished in ASD brains. Of particular interest is to explore the information transfer among brain areas that are part of a team to perform higher-order cognitive and social functions, which people with ASD particularly struggle with.

Understanding the information transfer, or the lack of it, between specific nodes in the brain may help uncover the neural bases of behavioral and social problems in ASD. It should be noted that only four previous studies have examined effective connectivity between brain regions in ASD (Bird et al., 2006; Wicker et al., 2008; Shih et al., 2010; Shen et al., 2012). These studies only permit limited inferences as they used a small number of regions and made prior assumptions about the underlying connectional architecture. This is because they used confirmatory methods such as dynamic causal modeling (Friston et al., 2003) and structural equation modeling (McIntosh and Gozales-Lima, 1994) in their studies. In contrast, the present study applies multivariate autoregressive (MVAR) modeling for obtaining Granger causality between a large number of brain regions. This is an exploratory technique which does not make any prior assumptions about the underlying connectional architecture. In addition, it is capable of obtaining condition-specific causal influences between a large number of brain regions using relatively shorter time series. According to the principle of Granger causality, the directional causal influence from time series *X* to time series *Y* can be inferred if past values of time series *X* help predict the present and future values of the time series *Y* (Granger, 1969). MVAR models have been used to characterize the predictive relationship between the time series from different brain regions in many previous studies (Roebroeck et al., 2005; Abler et al., 2006; Deshpande et al., 2008, 2009b; Sathian et al., 2011). But according to many recent studies, the spatial variability of the hemodynamic response is considered to be of vascular origin, and hence confounding the Granger causal estimates obtained from raw fMRI time series (David et al., 2008; Deshpande et al., 2010b). Removing the smoothing effect of the hemodynamic response function (HRF) will increase the effective temporal resolution of the signal in addition to accounting for the inter-subject and inter-regional variability of the HRF (Handwerker et al., 2004). This can be accomplished using blind hemodynamic deconvolution methods where in the underlying hidden neuronal variable for the fMRI time series can be estimated. We employed this approach in this study by deconvolving the hemodynamic response from fMRI time series using a Cubature Kalman filter (CKF) (Havlicek et al., 2011). Subsequently, these hidden neuronal variables were input into the MVAR model to obtain directional connectivity measures.

Investigating the directional interactions among brain areas in ASD could supplement functional connectivity findings, and potentially may serve as a neural signature for the disorder. Thus, connection abnormalities at anatomical, functional, and causal levels may be considered for potential diagnosis of ASD and/or to supplement the behavior-based diagnosis. However, such attempts will need to test and validate the diagnostic utility of connection abnormalities in ASD. Questions pertaining to diagnostic utility may be best answered through pattern classification analyses using sophisticated machine learning algorithms (Deshpande et al., 2010a; Weygandt et al., 2011; Shinkareva et al., 2013). In this regard, earlier studies have used pattern recognition and machine learning algorithms reliably in classification. Craddock et al. (2009) showed that by using resting state functional connectivity metrics as features in SVM based machine learning classifier, Major Depressive Disorder (MDD) patients were successfully distinguished from healthy controls. In another study, the treatment type provided to patients with MDD was accurately identified using SVM classifier based on the effective connectivity measures (Deshpande et al., 2009a). A pattern recognition approach using structural networks as biomarkers was proposed (Marquand et al., 2013) for classification of Parkinson's Disorder. This method of analysis accurately predicted the diagnosis in patients with Parkinson disorders. A study by Mirowski and colleagues (2009) showed that machine learning classifiers can be successfully used in prediction of seizures in patients with epilepsy. Given the success of pattern recognition and classification methods based on machine learning techniques in other fields and contexts, they could potentially prove to be useful to correctly identify participants with ASD after replication and fine tuning. In these lines, diagnostic information (although preliminary) has been obtained from even short fMRI BOLD sequences, such as characterization of subject age (Dosenbach et al., 2010), classification of dementia (Chen et al., 2011), and autism (Anderson et al., 2011; Murdaugh et al., 2012; Wang et al., 2012). For a neurodevelopmental disorder such as ASD, which is currently diagnosed solely by behavioral observation and in-person interviews by clinicians, classification by brain imaging signatures could be applied to gain more accurate (and perhaps earlier) diagnosis of the disorder. Classification studies have utilized a wide range of data sources to differentiate participants into ASD and TD groups, including functional connectivity (Anderson et al., 2011; Murdaugh et al., 2012; Wang et al., 2012), voxel based morphometry (Uddin et al., 2011; Calderoni et al., 2012), fMRI activation patterns (Coutanche et al., 2011), EEG (Duffy and Als, 2012), and DTI (Ingalhalikar et al., 2011). Yet, none of these methods are currently employed to diagnose the disorder. Issues remain regarding generalizability, such as whether the classification techniques can still be accurately applied to younger children. When other disorders also show functional connectivity and resting state abnormalities, such as schizophrenia (Lawrie et al., 2002; Meyer-Lindenberg et al., 2005; Garrity et al., 2007) and ADHD (Tian et al., 2006; Cubillo et al., 2010), it begs the question about the specificity of these metrics to ASD. However, notably, effective connectivity markers have not been used in classification of ASD individuals. In this regard, effective connectivity could be an additional data source utilized to add to classification of ASD participants, potentially providing sufficient information to serve as a biomarker for the disorder. In other words, effective connectivity could contribute significantly to the global connectivity-based neural characterization of ASD. Also, whereas traditional statistical analyses can uncover significant group differences in brain activation and connectivity, classification analyses can serve to identify brain imaging signatures which are not only able to separate or distinguish the groups, but also predict the group membership of a new subject.

In the current study we explored the causal influences between brain regions that may underlie the processing of theory-of-mind (ToM) in young adults with ASD and typically developing (TD) control participants. The original fMRI study on ToM was published earlier (Kana et al., 2012), reporting findings of brain activity, functional connectivity and white matter integrity. In the current study, we obtained causal connectivity between 18 brain regions activated in the ToM task in our previous publication (Kana et al., 2012). We used these causal connectivity weights along with the following metrics from our previous study—assessment scores, functional connectivity values and fractional anisotropy (FA) obtained from DTI data—as features for classification. We employed recursive cluster elimination to select important features and a support vector machine (SVM) classifier to classify participants into ASD and TD based on the entire feature set. This paper is novel in that it takes into consideration different aspects of brain connectivity, instead of a single index, to characterize the nature of brain functioning in individuals with ASD for classification purposes.

## Method

### Participants

Fifteen adolescents and young adults with high-functioning ASD (mean age: 21.14 years) and 15 age-and-IQ-matched individuals with typical development (TD) (mean age: 22.18 years) participated in this fMRI study. Functional connectivity, structural connectivity, behavioral data, and brain activation measures from the same participants were reported elsewhere (Kana et al., 2012). All participants were required to have an IQ of 80 or above measured by the Wechsler Abbreviated Scale of Intelligence (WASI). The participants with ASD were recruited from the University of Alabama ASD Clinic and surrounding service providers. The study was approved by the Institutional Review Board of the University of Alabama at Birmingham, and all participants provided informed consent for their participation in the study. Participants with ASD had received a previous diagnosis of an ASD based on Autism Diagnostic Interview (ADI-R) symptoms, and Autism Diagnostic Observation Schedule (ADOS). Eight of the 15 ASD participants in this study had received a diagnosis of Asperger's Disorder. The TD participants were recruited through newspaper advertisements and through the University of Alabama at Birmingham's Psychology 101 course subject pool. They were screened through a parent-report (for participants younger than 18 years) or self-report history questionnaire to rule out neurological disorders, such as ASD, ADHD, or Tourette's Disorder, that could potentially confound the results. All participants completed the Autism Spectrum Quotient (AQ) questionnaire (Baron-Cohen et al., 2001b), and the Reading the Mind in the Eyes (RME) test (Baron-Cohen et al., 2001a). Demographic information about the participants is shown in Table 1.

**Table 1:** **Demographic information of the ASD and TD control participants**.

	**Autism**					**Control**						
	**N = 15**					**N = 15**					**Group difference**	
	**Mean**	**Range**	***SD***	**Median**	**IQR**	**Mean**	**Range**	***SD***	**Median**	**IQR**	***t*-value**	***p*-value**
Age	21.14	16–29	0.99	20.08	5	22.28	16–34	1.08	21.83	3.3	0.77	0.44
VIQ	104.8	74–139	5.02	102	31	113.93	98–127	2.2	116	14	1.66	0.11
PIQ	107.7	73–129	4.33	106	25	107.2	89–124	2.48	108	6	0.11	0.92
FSIQ	106.93	80–140	4.84	105	35	112	96–128	2.24	113	13	0.94	0.35
MIE	19.07	15–24	0.7	20	4	21.6	18–24	0.55	22	4	2.84	0.01
AQ	26.5	9–38	2.04	29	12	14.06	4–22	1.45	14	9	5.47	<0.001

### Experimental paradigm and imaging parameters

The stimuli consisted of a series of black and white comic strip vignettes (adapted from Brunet et al., 2000) depicting scenarios that demand either a physical causal attribution or an intentional causal attribution to arrive at a logical ending. The first part of the vignette was presented for 5 s and the participants' task was to choose a logical ending to the story from the three choices in the second panel presented for 6 s. The entire vignette remained on the screen for a total of 11 s. The experiment was designed in an event-related format. All data were collected using a Siemens 3.0 Tesla Allegra head-only scanner (*Siemens* Medical Inc., Erlangen, Germany). For functional imaging, a single-shot gradient-recalled echo-planar pulse sequence was used for rapid image acquisition (*TR* = 1000 ms, *TE* = 30 ms, flip angle = 60 degrees). Seventeen adjacent oblique-axial slices were acquired in an interleaved sequence with 5 mm slice thickness, 1 mm slice gap, a 24 × 24 cm^2^ field of view (FOV), and a 64 × 64 matrix, resulting in an in-plane resolution of 3.75 × 3.75 × 5 mm^3^. More information on the experimental paradigm and imaging parameters for the 3D MPRAGE structural MRI data and diffusion weighted echo-planar imaging data can be found in Appendix A (for further details, please refer to Kana et al., 2012).

### Data analyses

#### Head motion correction and regions of interest (ROI) definition

Within-group brain activation was examined for the whole group (ASD + TD) of participants (see Kana et al., 2012). Functional ROIs were defined on the group activation map for the whole group (ASD + TD) for the contrast (Intentional Causality + Physical Causality) vs. Fixation, so that it best represented the study. Because head motion can impact connectivity analyses (Satterthwaite et al., 2012; Van Dijk et al., 2012), a conservative threshold of 0.5 mm was set for head motion in any direction. In addition, the root mean square (RMS) values of head motion were measured in three translational directions (*x*, *y*, and *z*) and three rotations (pitch, roll, and yaw) for each individual participant in the study (see Appendix B Table B1). We examined group differences in head motion on this data using a Mann-Whitney *U* Test, which is a non-parametric test and may be more appropriate in case assumptions about normality of sample distributions are not met.

Eighteen ROIs were identified: supplementary motor area (SMA), left and right inferior frontal gyrus (LIFG, RIFG), left and right precentral cortex (LPRCN, RPRCN), left and right middle temporal gyrus (LMTG, RMTG), right superior temporal gyrus (RSTG), left and right inferior parietal lobule (LIPL, RIPL), left and right fusiform gyrus (LFFG, RFFG), left and right superior parietal lobule (LSPL, RSPL), left and right middle occipital gyrus (LMOG, RMOG), and left and right temporal parietal junction (LTPJ, RTPJ). A sphere was defined for each cluster (with a radius ranging from 8 to 12 mm) that best captured the cluster of activation in the contrast map for each group. The radius was selected to specifically encompass as much of the activation cluster as possible, without including surrounding (not significantly activated) areas. Selecting ROIs of the same radius or utilizing anatomically defined ROIs may entail those ROIs not encompassing the entire cluster of activation, or may include tissue that is not significantly active for the task. As a result, extracting time courses from ROIs defined in these ways may result in time series variability that does not reflect the cognitive task being processed.

#### The effective connectivity model

Let *l* fMRI time series be represented as *X*(*t*) = [*x*_1_(*t*)*x*_2_(*t*)… *x*_*l*_(*t*)]. Below, we present a model linking observed fMRI time series to underlying latent neuronal variables. A dynamic state-space model can be described as follows.

(1) $${\tilde{h}}_{T}^{l}=\left[\begin{array}{l} h_{T}^{l}\\ u_{T}^{l}\\ \theta_{T}^{l} \end{array}\right]=\left[\begin{array}{c} f(h_{T-1}^{l},u_{T-1}^{l},\theta_{T-1}^{l})\\ u_{T-1}^{l}\\ \theta_{T-1}^{l} \end{array}\right]+\left[\begin{array}{l} P_{T-1}^{l}\\ Q_{T-1}^{l}\\ R_{T-1}^{l} \end{array}\right]$$

Where *h* is the hidden neuronal state variable, *u* is the exogenous input and θ represents the HRF parameter variables. ***f*** is the function which links the current neuronal state to the previous neuronal states, exogenous inputs and parameters. The subscript *T* indicates continuous time and the superscript *l* indicates the number of time series in the model. *P*, *Q*, and *R* are the zero mean Gaussian state noise vectors. The observation equation links the state to observation variables as given below.

(2) $$x_{l}(t)=m({\tilde{h}}_{t}^{l})+\varepsilon_{t-1}$$

where *ε* is the measurement noise, *t* is discrete time and ***m*** is the measurement function which links the state variables to measurement variables. The exogenous inputs *u*, which is the experimental boxcar function, and *x*_*l*_(*t*) are the inputs to the model. As demonstrated before, using the CKF (Havlicek et al., 2011), the hidden neuronal variables can be estimated successfully. The CKF performs very efficient joint estimation of the hidden neuronal state variables and parameters. In addition, since Eq. 1 represents a continuous time model, the neuronal variables can be estimated with a highly improved temporal resolution up to 10 times smaller than the TR. When the hidden neuronal state variables *h*_*l*_(*t*) are input into the MVAR model, we get the following equation.

(3) $$\begin{array}{l} \left[\begin{array}{c} h_{1}(t)\\ h_{2}(t)\\ \cdot \\ \cdot \\ h_{l}(t) \end{array}\right]=\left[\begin{array}{cccc} 0 & a_{12}(0) & \cdots & a_{1l}(0)\\ a_{21}(0) & 0 & & a_{2l}(0)\\ \begin{array}{l} \cdot \\ \cdot \end{array} & \begin{array}{l} \cdot \\ \cdot \end{array} & \begin{array}{l} 0 \end{array} & \begin{array}{c} \cdot \\ \cdot \end{array}\\ a_{l1}(0) & a_{l2}(0) & \cdots & 0 \end{array}\right]\times \left[\begin{array}{c} h_{1}(t)\\ h_{2}(t)\\ \cdot \\ \cdot \\ h_{l}(t) \end{array}\right]\\ \;+\sum_{j=1}^{p}\left[\begin{array}{cccc} a_{11}(j) & a_{12}(j) & \ldots & a_{1l}(j)\\ a_{21}(j) & a_{22}(j) & & a_{2l}(j)\\ \begin{array}{l} \cdot \\ \cdot \end{array} & \begin{array}{l} \cdot \\ \cdot \end{array} & & \begin{array}{l} \cdot \\ \cdot \end{array}\\ a_{l1}(j) & a_{l2}(j) & \ldots & a_{ll}(j) \end{array}\right]\times \left[\begin{array}{c} h_{1}(t-j)\\ h_{2}(t-j)\\ \cdot \\ \cdot \\ h_{l}(t-j) \end{array}\right]+\left[\begin{array}{c} e_{1}(t)\\ e_{2}(t)\\ \cdot \\ \cdot \\ e_{l}(t) \end{array}\right] \end{array}$$

where *p* is the model order estimated by the Akaike/Bayesian information criterion (Deshpande et al., 2009b), *a* represent the model coefficients and *e* represents the error of the MVAR model. From the above equation it can be observed that *a*(0) represents the instantaneous influences between the time series, and the Granger causal influences between them is indicated by *a*(*j*), *j* = 1 …. *p*. Both terms are used in the model because including both instantaneous and causal terms in the model minimizes the “leakage” of instantaneous correlation into causality (Deshpande et al., 2010c). The multivariate model we have used is less sensitive to the effects of missing variables than the traditionally used pairwise bivariate models (Kuś et al., 2004). Also, since we included all 18 regions which were activated in the effect of interest, it guaranteed to a certain level that all regions involved in the task were indeed included in the model.

#### Effective connectivity analysis

Mean time series from 18 activated regions were obtained for each of the 15 participants with ASD and the 15 typical control participants. Using the boxcar function corresponding to “intentional causality” as the exogenous input, hidden neuronal variables corresponding to normalized mean fMRI time series were obtained and input into the MVAR model. The Granger causal relationships between the 18 regions for each participant (ASD and TD) were obtained. The number of coefficients in the MVAR model is equal to *k*^2^*p* (*k* is the number of time series and *p* is the model order) (Kuś et al., 2004). This must be smaller than the number of time points in each time series. We had 18 ROI time series, each of length 460. Since we used a first order model, *k*^2^*p* = 324 which is less than 460. Therefore, we were able to estimate the model.

#### Classification using support vector machine

The statistical separation of neural signatures (e.g., *t*-test) does not guarantee generalizability or predictive power of those signatures for diagnosis. Therefore, in this study, we also used machine learning approaches for identification of metrics which can accurately classify individuals with ASD from individuals with typical development. A Recursive Cluster Elimination based Support Vector Machine (RCE-SVM) (Deshpande et al., 2010a) was used in this study to classify the participants based on granger causal path weights between the 18 ROIs, functional connectivity z-scores for all pairs of the 18 ROIs, assessment scores (AQ and RME scores) and FA values for the white matter tract extending into the temporal lobe as the input features. The functional connectivity, assessment and DTI FA values were obtained from our prior study (Kana et al., 2012).

**Figure 1:**
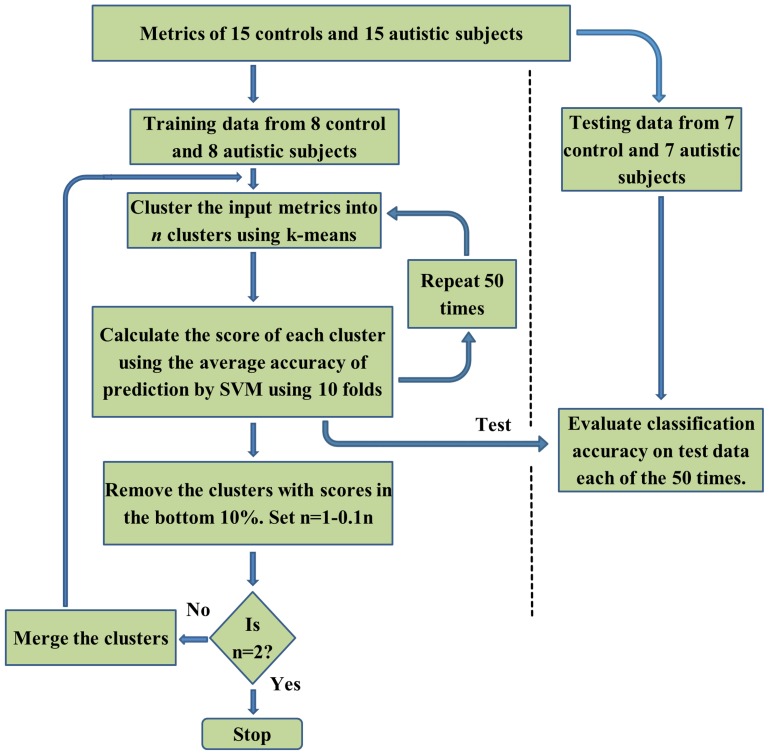
**A flow-chart depicting the Recursive Cluster Elimination based Support Vector Machine (RCE-SVM) procedure**.

Our choice of SVM for classification was motivated by its wide applicability as a machine learning approach (Vapnik, 1995) for classification in many different fields (Wang, 2005). Previous studies have demonstrated that using discriminatory features enhances SVM classification (Craddock et al., 2009). Therefore, to enhance the performance of the SVM classifier, filtering and wrapper methods for feature selection have been used. Filtering methods are based on extraction of features that are statistically different between classes. They can be extracted using statistical tests such as a *t*-test. The wrapper approach is based on iteratively eliminating features to minimize the prediction error. RCE is one of the wrapper methods that is an iterative process were the feature selection and classification steps are embedded with each other. The main steps of the RCE-SVM algorithm, shown in the flowchart in Figure 1, are the cluster step, the SVM scoring step and the RCE step. Initially, the features that were input into the classifier were divided into training and testing data sets. Fifty such splits were carried out in order to ensure the generalizability of the results. In the clustering step, k-means algorithm (Yang et al., 2003) was used to cluster the training data into *n* clusters. The number of clusters was first set to the number of features, and was progressively decreased by one until there were no empty clusters. The *n* obtained by this iteration served as the initial *n* for the RCE-SVM loop.

In the SVM scoring step, each cluster was scored based on its ability to differentiate the two categories by applying linear SVM. In order to rate the clusters, the training data was randomly partitioned into 10 non-overlapping subsets of equal sizes (10 folds). Using 9 subsets, the linear SVM was trained and performance was calculated using the remaining subset. Different possible partitions were taken into account by repeating the clustering and cross validation procedure 50 times. For each of these 50 repetitions, the classification accuracy of SVM was ascertained using the test data. The average value of this accuracy, taking into account the repetitions and all the folds was assigned as the score of the cluster. The bottom 10% of low score clusters were eliminated in the RCE step. The remaining features were merged and the value of *n* was decreased by 10% and the cluster step, the SVM scoring step and the RCE step were repeated again in an iterative manner. After each iteration, the performance of the classifier was assessed using the testing data and lesser number of features compared to the earlier iteration. When the number of clusters was equal to two, the procedure was stopped. Complete separation of testing and training data in this algorithm eliminates bias in performance accuracy (Kriegeskorte et al., 2009). The accuracy at every RCE-SVM loop was calculated as a mean value of accuracy obtained over 50 repetitions of each loop and each train-test split, using the feature clusters of test data available at the corresponding loop and split. The statistical significance of mean accuracies was calculated by estimating the *p*-values of a binomial null distribution B(η,ρ), η being the number of participants and ρ is the probability of accurate classification as in previous studies (Pereira et al., 2009). Only accuracies whose *p*-values were less than 0.05 after correcting for multiple comparisons using Bonferroni method were considered as statistically significant.

The causal connectivity weights obtained from the MVAR model, the behavioral assessment scores, the functional connectivity z-scores for each ROI pair, and DTI FA metrics for each of the 30 subjects (15 ASD and 15 TD) were input into the RCE-SVM classifier to determine the accuracy with which the classifier can predict a novel subject's group membership (autism or control).

## Results

The main results of this study are summarized as follows: (1) The effective connectivity path weights were able to successfully classify participants by diagnosis with 95.9% accuracy. These path weights were the most discriminative features among all the different metrics used in classification; (2) Effective connectivity paths most important for classification were significantly reduced (*p* < 0.05) in ASD participants compared to typical control participants; and (3) The paths that were among the top ranked features in the classification analysis were found to be negatively correlated with the AQ and positively correlated with the RME test scores.

**Figure 2:**
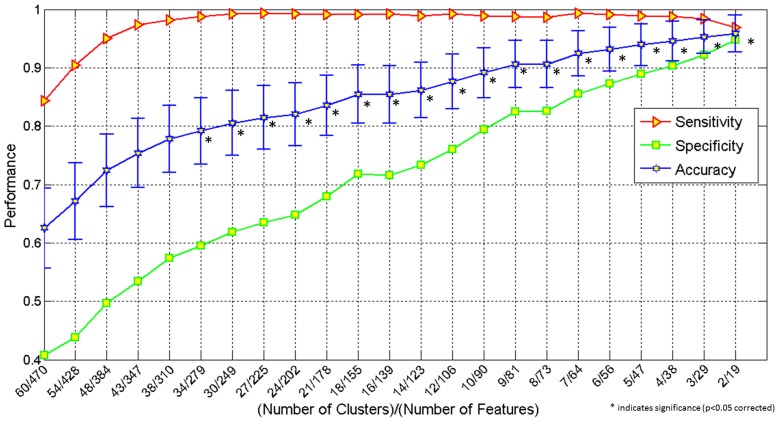
**Graph showing classification accuracy, sensitivity and specificity obtained by simultaneously using the following features: behavioral scores, functional connectivity, effective connectivity and fractional anisotropy obtained from DTI**. The X-axis shows number of clusters/number of features and the Y-axis, the performance (classification accuracy, sensitivity and specificity). ^*^indicates significance (*p* < 0.05 corrected).

The first set of results pertains to a pattern classification analysis involving several indices of connectivity (functional connectivity, effective connectivity, white matter integrity) and performance accuracy in this ToM task. In this analysis, utilizing 2 feature clusters comprised of 19 metrics, the classification accuracy reached a maximum accuracy of 95.9% (specificity 94.8%, and sensitivity 96.9%). It should be noted that all of the 19 features were effective connectivity paths. Figure 2 demonstrates the increase in performance of classification with decreasing number of features (and removal of uninformative features). The *p*-values for all the accuracy values shown in Figure 2 can be seen in Table 2.

**Table 2:** **Classification accuracy values and the corresponding *p*-values obtained at each step of the RCE algorithm**.

**Accuracy**	***p*-Value**
0.625	0.100244
0.672	0.049369
0.724	0.008062
0.754	0.002611
0.778	0.002611
0.792	0.000715
0.805	0.000715
0.815	0.000715
0.82	0.000162
0.835	0.000162
0.855	2.97 × 10^−05^
0.854	2.97 × 10^−05^
0.862	2.97 × 10^−05^
0.876	2.97 × 10^−05^
0.892	4.22 × 10^−06^
0.906	4.22 × 10^−06^
0.906	4.22 × 10^−06^
0.925	4.34 × 10^−07^
0.932	4.34 × 10^−07^
0.939	4.34 × 10^−07^
0.945	4.34 × 10^−07^
0.953	2.89 × 10^−08^
0.959	2.89 × 10^−08^

**Figure 3:**
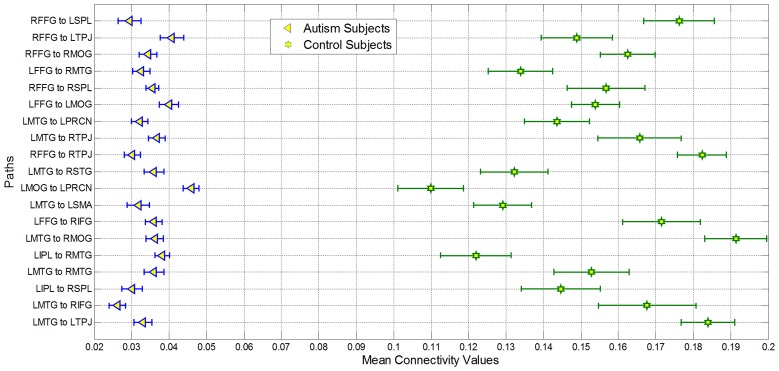
**Mean of nineteen paths which was most important for giving maximum classification accuracy for autism and control groups**. All paths had significantly decreased connectivity (*p* < 0.05 corrected using Bonferroni method for 18 paths; for one of the paths *p* < 0.05 uncorrected) in the Autism group as compared to controls. The bars represent standard errors.

**Figure B1:**
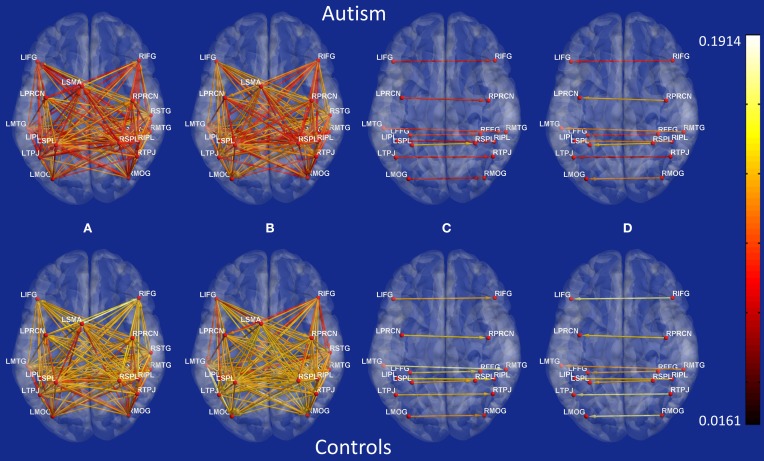
**Connectivity maps showing Granger causality path weights for all possible connections between 18 ROIs**. Top: Autism, Bottom: Controls. **(A)** posterior to anterior paths, **(B)** anterior to posterior paths, **(C)** left to right paths, **(D)** right to left paths.

**Figure B2:**
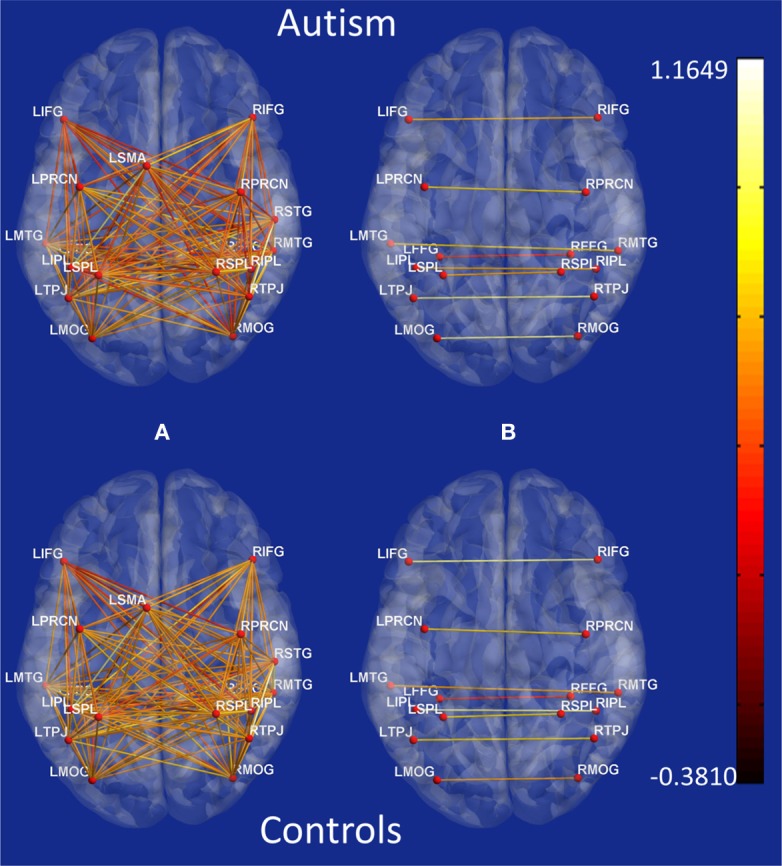
**Connectivity maps showing *z*-scores of functional connectivity path weights obtained using Pearson's correlation for all possible connections between 18 ROIs**. Top: Autism, Bottom: Controls. **(A)** anterior–posterior paths, **(B)** bilateral paths.

Second, the causal connectivity weights of the 19 paths which led to maximum accuracy of 95.9% showed clear separation between participants with autism (blue) and typical control participants (green) as shown in Figure 3, with these paths showing significantly (*p* < 0.05 corrected using Bonferroni method for 18 paths; for one of the paths *p* < 0.05 uncorrected) weaker connectivity in participants with ASD compared to TD controls. Many of these connections are between regions that are part of the social brain network (LTPJ, RTPJ, LFFG, RFFG, LMTG, RMTG, RIFG) which may prove critical in accomplishing the ToM task used in this study. It is noteworthy that there may be other paths which are significantly different between the groups. Here, we restrict ourselves to finding the statistical separation of features which have the highest ability for predicting the diagnosis of a given subject. We do so primarily because we are interested in features with predictive ability rather than those which just “differ” between the groups. Please refer to Appendix B Figures B1, B2 in order to gain a qualitative understanding of the functional and effective connectivity paths, respectively, between all 18 ROIs in both ASD and TD groups.

**Figure 4:**
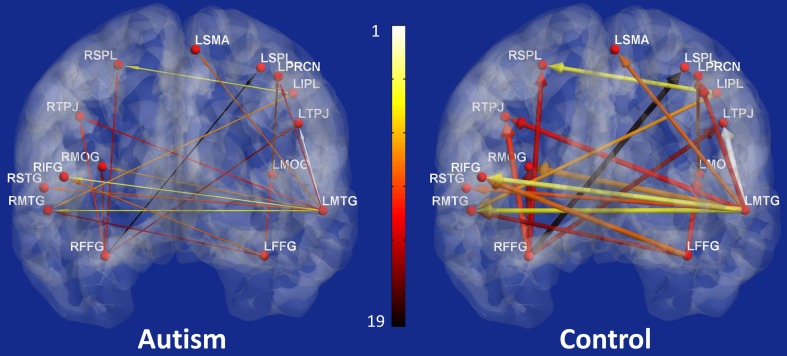
**The nineteen paths whose effective connectivity values were top-ranked features for classification of the two groups (Autism and Controls) with the maximum accuracy (Left panel: participants with autism; and right panel: control participants)**. The width of the arrows represents the path strength and the color of the path indicates its rank obtained during classification with 1 being the most significant and 19 being the least significant.

The 19 effective connectivity paths which were most important in classification are shown in Figure 4. The left panel shows these paths in ASD participants and the right panel in control participants. The width of the arrows illustrates the path weight in the corresponding group and the color represents the rank of the path obtained during classification.

Third, a correlation analysis was also performed between the features that were ranked highest in classification and gave rise to maximum accuracy, and assessment scores (AQ and RME). Given that the top-ranked features are not guaranteed to have normal distribution, we used Spearman's non-parametric correlation method to determine whether the top-ranked features were correlated with behavior. This analysis (including all participants in the study) revealed a significant negative correlation between several effective connectivity paths and the AQ scores as well as a significant positive correlation between effective connectivity paths and RME scores (see Table 3 for specific paths, correlation and *p*-values). These results suggest that as autism symptom severity increased, the effective connectivity of the top-ranked paths decreased; and as the theory-of-mind ability increased, effective connectivity of the top-ranked paths also increased. This provides a second-level test of the behavioral relevance of the top-ranked paths, which is to be expected given the fact that diagnosis was based on behavioral symptoms. As a cautionary note, these results should not be construed as a general discovery regarding brain connectivity features in autism which correlate with behavioral symptoms.

**Table 3:**
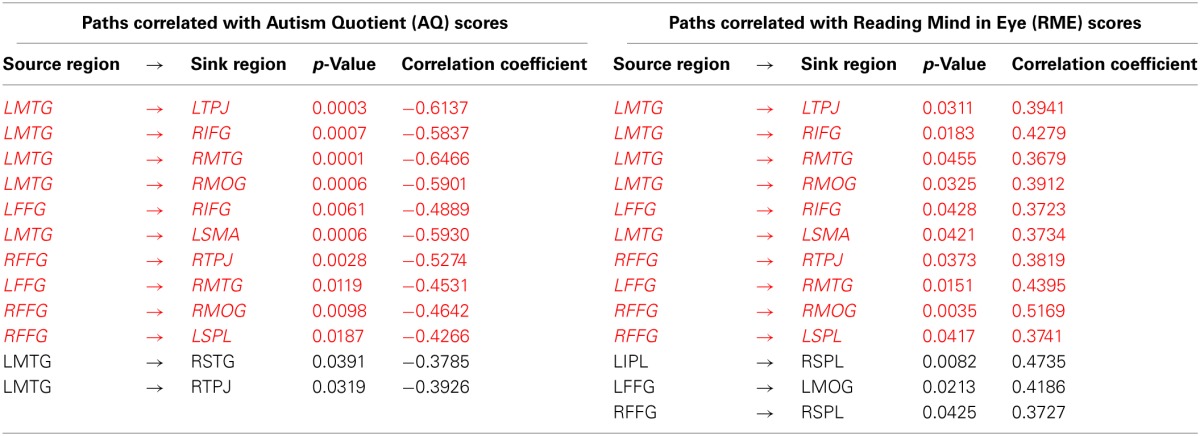
**Paths correlated with Autism Quotient (AQ) scores and Reading Mind in Eye (RME) scores**.

The paths in red were correlated with both AQ and RME.

Neuroimaging data, especially brain connectivity analyses are prone to be influenced by head motion and signal quality. We conducted several different measures to make sure that our data and the reported results were not influenced by quality related issues. First, the root mean square (RMS) values for each subject and each head motion parameter were obtained (see Appendix B Table B1). The RMS values were then submitted to a non-parametric Mann-Whitney *U* test, which also revealed no significant difference in motion in x [*U*_(28)_ = 66, *Z* = −1.929, *p* = 0.06], y [*U*_(28)_ = 93, *Z* = −0.809, *p* = 0.42], and z [*U*_(28)_ = 96, *Z* = −0.684, *p* = 0.49] translational directions. Nor was there a significant group difference in rotation in pitch [*U*_(28)_ = 68, *Z* = −1.846, *p* = 0.06], roll [*U*_(28)_ = 107, *Z* = −0.228, *p* = 0.82], and yaw [*U*_(28)_ = 93, *Z* = −0.809, *p* = 0.42]. These results indicate that there were no statistical differences in head motion between the two groups, assuming a *p*-value threshold of 0.05. However, there was a non-significant trend (*p* = 0.06) for translation in x direction and the degree of rotation in pitch to differ between the groups.

Further, we obtained the mean value of frame wise displacement (FD) for each subject as a quality control (QC) metric and investigated whether they correlated with any of the 19 top-ranked paths obtained from classification across the entire sample. The instantaneous motion of the head was expressed as a scalar quantity using the formula, *FD*_*i*_ = |Δ*d*_*ix*_| + |Δ*d_iy_*| + |Δ*d_iz_*| + |Δα_*i*_| + |Δβ_*i*_| + |Δγ_*i*_|, where Δ*d_ik_* = *d*_(*j* − 1)*k*_ − *d_ik_* and *k* is any of the 3 translational parameters (*x*, *y*, *z*) or rotational parameters (α, β, γ). We converted the rotational displacements from degrees to millimeters by calculating displacement on the surface of a sphere of radius 50 mm, assuming that the approximate mean distance from the center of the head to the cerebral cortex is 50 mm. The above procedure of calculating FD and correlating its mean with connectivity metrics obtained from individual subjects has been recommended recently for either confirming or ruling out the influence of head motion on connectivity measures (Power et al., 2012; Satterthwaite et al., 2012; Van Dijk et al., 2012; Satterthwaite et al., 2013; Yan et al., 2013). The QC-connectivity Spearman's correlations and corresponding *p*-values indicating their statistical significance are shown in Table 4. It is evident that none of the QC-connectivity correlations were statistically significant (*p* > 0.05). Given these evidence, any significant group differences for imaging metrics was probably not due to head motion. We did not use the scrubbing method described in Power et al. (2012), where removal of certain parts of the time series (scrubbing) creates an artificial discontinuity in the data. This may not be a problem while using Pearson's correlation coefficient as zero-lag synchronization in the data does not depend on the temporal ordering in the data as long as the correspondence between the variables being examined is preserved. However, other methods which are sensitive to temporal ordering in the data cannot use scrubbing. Granger causality is one such method which is indeed sensitive to temporal ordering in the data and hence we did not use scrubbing.

**Table 4:** **The Spearman's correlation between mean frame wise displacement (our quality control metric) and the Granger causality weights for the top ranked 19 paths**.

**Source region**	→	**Sink region**	***p*-Value**	**Correlation coefficient**
LMTG	→	LTPJ	0.869	−0.031
LMTG	→	RIFG	0.958	0.010
LIPL	→	RSPL	0.984	−0.003
LMTG	→	RMTG	0.701	−0.073
LIPL	→	RMTG	0.547	0.115
LMTG	→	RMOG	0.856	−0.035
LFFG	→	RIFG	0.309	0.192
LMTG	→	LSMA	0.741	−0.063
LMOG	→	LPRCN	0.827	0.042
LMTG	→	RSTG	0.725	−0.067
RFFG	→	RTPJ	0.599	0.099
LMTG	→	RTPJ	0.827	−0.042
LMTG	→	LPRCN	0.665	−0.083
LFFG	→	LMOG	0.702	−0.073
RFFG	→	RSPL	0.322	−0.187
LFFG	→	RMTG	0.623	0.094
RFFG	→	RMOG	0.404	−0.158
RFFG	→	LTPJ	0.703	−0.073
RFFG	→	LSPL	0.866	0.032

The paths shown in the table are ordered according to the rank obtained during classification with 1 being the most significant and 19 being the least significant (first path is Rank −1 and the last path is Rank −19).

Differences in signal to noise ratio (SNR) can also impact Granger causality estimates (Nalatore et al., 2007) when the SNR is low. On the other hand, when SNR = 2, which is typically the case for task-based fMRI, we have previously showed using simulations that Granger causality estimates are accurate in the absence of hemodynamic variability (which is the case here since we deconvolved the hemodynamic response) (Deshpande et al., 2010b). We calculated effective SNR of the deconvolved fMRI time series by estimating the variance of the entire deconvolved signal, i.e., the hidden neuronal variable, and divided it by the variance of the deconvolved signal during non-stimulation phases. We then populated the SNRs of each ROI in autism and control groups to two different samples and performed a non-parametric Wilcoxon ranksum to find statistical differences. The SNR was significantly higher (*p* < 0.05, *z*-value = 20.1) in the ASD group (SNR = 4.13 ± 0.01) as compared to the control group (SNR = 3.2 ± 0.03). The SNRs for both groups were high enough so that SNR differences between the groups will not impact Granger causality. SNR has an impact on Granger causality only when the SNR is low.

## Discussion

The goals of this study were: (1) to investigate effective connectivity among brain areas during intentional causal attribution in ASD and (2) to utilize machine learning techniques to classify participants based on effective connectivity weights from this study, and behavior assessment scores, functional connectivity, and fractional anisotropy obtained from DTI data from our previous study (Kana et al., 2012). Using SVM based classification, we found that the causal connectivity path weights had the highest discriminative power to separate groups by diagnosis with high accuracy. It was uncovered that the top-ranked causal connectivity paths were also significantly weaker between social brain regions in young adults with ASD as compared to their TD peers and correlated with the ASD symptom severity (AQ) scores and theory-of-mind ability as measured by the RME test.

An application of characterizing brain connectivity patterns is to test whether such patterns can differentiate individuals with ASD from typically developing control participants such that the diagnostic label of a new participant can be determined based on imaging data. Thus, in this study we conducted a classification analysis using the effective connectivity measures, functional connectivity values, fractional anisotropy obtained from DTI data and the causal attribution task performance scores to get a fair assessment of which metric possesses the highest discriminative power. A maximum classification accuracy of 95.9% was obtained with 2 clusters and 19 features, all of these being effective connectivity paths. These results suggest that significantly weaker causal influence between brain regions during ToM processing in ASD is sufficient to separate adults with ASD from typical control participants. The discriminative patterns found in this study using SVM may have clinical applications in the long-run. Accurate separation of ASD adults from TD peers may provide potential value for clinicians, particularly in cases when behavioral observation and clinical interviews are not sufficient enough to determine a diagnosis. The key finding of differences in the causal influence of brain regions for ToM in ASD in this study adds to the relatively limited literature on effective connectivity in ASD. In addition, while previous studies explored effective connectivity in ASD during language processing, facial and emotional processing, and imitation, the current study examined effective connectivity in the context of a ToM task, which has not been studied in ASD to date. The current study expands what we know about inferring mental states in ASD, and provides insight into the causal relationships of brain regions during ToM processing. In addition, this study, to our knowledge, is the first to use effective connectivity measures for classification purposes in ASD. While this method will require some fine tuning, validation in a larger sample, and replication through multiple studies to be applied within clinical settings, the causal relationships between brain areas related to ToM holds promise for separating individuals with ASD from typical controls or from other disorders. Nevertheless, the current study marks the first attempt at using effective connectivity measures as inputs for a classification analysis of ASD subjects, therefore marking the first step in the direction of more accurate classification of the disorder.

Weaker effective connectivity of the 19 top-ranked paths found in participants with ASD in this study involved paths and regions that are found to be part of the social brain network. Several nodes, such as the TPJ, MTG, RIFG, IPL, FFG, and SMA have been associated with processing theory-of-mind, face processing, and the mirror neuron system. These findings are in line with previous studies of effective connectivity in ASD (Wicker et al., 2008; Shih et al., 2010). Our results also include significant functional alterations in social brain and visuospatial brain regions (e.g., TPJ, IFG, IPL, FFG, etc.) seen previously in functional connectivity findings (Kana et al., 2006, 2009, 2012; Just et al., 2007; Koshino et al., 2008; Mason et al., 2008), suggesting some consistency in disrupted connectivity across different modalities of connectivity and providing further support for disrupted connectivity accounts of ASD (Just et al., 2004; Kana and Just, 2011; Schipul et al., 2011; Kana et al., 2012). The findings here supplement the functional connectivity results in our previous study utilizing the same ToM stimuli, where ASD participants displayed significantly reduced functional connectivity between temporal and frontal regions, and weaker connectivity between networks made up of ventral premotor regions and TPJ (Kana et al., 2012). Our results in the current study further these previous findings by illustrating the directionality of connectivity. We found that, for ToM processing in TD participants, significantly stronger (compared to ASD group) causal connections existed among the 19 top-ranked paths which included the nodes that are associated with social cognition. So, here we find that the critical regions of the social brain are not as well coordinated with others, that they should be sharing information with, in participants with ASD. This lack of synchrony and reduced flow of information may represent a critical problem of bandwidth (maximal rate of data transfer supported by a communication channel) in ASD, where some information is getting by, but at a much lower rate than what would be needed for complex ToM connections (Just et al., 2012).

In a correlation analysis using assessment measures and effective connectivity paths for the entire sample of participants, we found the paths that were among the top ranked features in the classification analysis were correlated with AQ and RME scores. While the AQ showed significant negative correlation, the RME showed significant positive correlation with connectivity paths. Similarly, participants with better ToM skills had stronger effective connectivity during this causal attribution task. It should be noted that most of these connection paths involved information transfer to different regions mainly from the temporal lobe (LMTG, and bilateral FFG). While FFG has been associated with face processing and processing socially salient stimuli (Schultz, 2005), middle and superior temporal areas have been found to be involved in social cognition, especially in taking intentional stance, as seen in the current study, on social scenarios (Mosconi et al., 2005). The correlations found in our study reveal how social abilities such as ToM skill can influence information transfer in the brain. In addition, it also points out that severe autism symptoms may have a neural basis in reduced causal brain connectivity from the temporal lobe. As noted earlier, the correlation analysis was performed across the entire sample and we restricted it to the top-ranked 19 paths because we feel that the covariance of a brain imaging based metric with a behavioral assessment score is clinically meaningful only if the imaging metric under consideration has the power to predict the diagnostic label of a new subject. Therefore, there may very well be other connectivity paths in the brain which may be correlated with behavior (but which lack the discriminative ability) which we have not discussed here.

In conclusion, this study provides preliminary evidence to support a hypothesis that metrics based on directional brain connectivity obtained from a task engaging social brain areas may provide highly discriminative features for predicting whether a given subject has ASD or not. Studies involving larger sample size, and replication of these findings across multiple studies would be required to fully test the extent of this hypothesis and investigate its clinical implications.

### Conflict of interest statement

The authors declare that the research was conducted in the absence of any commercial or financial relationships that could be construed as a potential conflict of interest.

## Appendix A

### Image acquisition

Structural Imaging: Acquisition of initial high-resolution T1-weighted scans was done using a 160-slice 3D MPRAGE volume scan with *TR* = 200 ms, *TE* = 3.34 ms, flip angle = 78, field of view (FOV) = 25.6 cm, matrix size = 256 × 256 and slice thickness = 1 mm.

Diffusion tensor imaging: A diffusion-weighted, single-shot, spin-echo, echo-planar imaging (EPI) sequence (*TR* = 4400 ms, *TE* = 85 ms, bandwidth = 1860 Hz/voxel, FOV = 240 mm and 128 × 128 matrix size, resulting in an in-plane resolution of 1.87 × 1.87 × 3 mm) was used to collect the images. Thirty-two 3 mm thick slices were imaged (no slice gap) with no diffusion weighting (*b* = 0 s/mm^2^) and with diffusion weighting (*b* = 1000 s/mm^2^) gradients applied in 12 orthogonal directions. Twenty-four images of each slice by gradient direction combination were acquired and averaged to produce the final diffusion imaging data set.

### Data analysis

The brain activation data were analyzed using Statistical Parametric Mapping (SPM8) software (Wellcome Department of Cognitive Neurology, London, UK). Images were corrected for slice acquisition timing, motion-corrected, normalized to the Montreal Neurological Institute (MNI) template, resampled to 2 × 2 × 2 mm voxels, and smoothed with an 8-mm Gaussian kernel to decrease spatial noise. We performed statistical analysis on individual and group data by using SPM8's implementation of the general linear model (Friston et al., 1995). Within-group activation was analyzed for the ASD group, TD group, and for the whole group (ASD + TD) of participants. Activation data was analyzed for all trials with separate regressors defined for intentional causality, physical causality, and fixation baseline conditions. The within-group analyses used a cluster size of 80 mm^3^ determined by 10,000 Monte Carlo simulations at an uncorrected *p* value of 0.001. According to Lieberman and Cunningham (2009), simulations can implicate cluster size thresholds that produce the best balance between Type I and Type II error. The between-group analyses used a cluster threshold of 10 contiguous voxels at an uncorrected *p* value of 0.005, as the effects did not survive a more conservative statistical threshold.

ROIs were defined on the group activation map for the whole group (ASD + TD) for the contrast Intention + Physical vs. Fixation, so that it best represents the study. Eighteen ROIs were identified: supplementary motor area (SMA), left and right inferior frontal gyrus (LIFG, RIFG), left and right ventral premotor cortex (LPMv, RPMv), left and right middle temporal gyrus (LMTG, RMTG), right superior temporal gyrus (RSTG), left and right inferior parietal lobule (LIPL, RIPL), left and right fusiform gyrus (LFFG, RFFG), left and right superior parietal lobule (LSPL, RSPL), left and right middle occipital gyrus (LMOG, RMOG), and left and right temporal parietal junction (LTPJ, RTPJ). A sphere was defined for each cluster (with a radius ranging from 8 to 12 mm) that best captured the cluster of activation in the contrast map for each group. The activation time-course extracted for each participant over the activated voxels within the ROI originated from the normalized and smoothed images that were low-pass filtered and had the linear trend removed.

## Appendix B

**Table B1:** **Root mean square values of head motion**.

	**Translation**			**Rotation**		
	***X***	***Y***	***Z***	**Pitch**	**Roll**	**Yaw**
**ASD**						
1	0.1456	0.1073	0.2415	0.0024	0.0013	0.0020
1	0.1234	0.0910	0.1028	0.0010	0.0013	0.0017
1	0.1009	0.1698	0.2096	0.0036	0.0023	0.0012
1	0.1521	0.2020	0.1704	0.0013	0.0015	0.0009
1	0.1216	0.1725	0.2679	0.0031	0.0014	0.0015
1	0.1090	0.1473	0.3443	0.0037	0.0011	0.0018
1	0.0639	0.0863	0.2077	0.0027	0.0013	0.0005
1	0.1082	0.0714	0.2311	0.0024	0.0012	0.0014
1	0.2747	0.2213	0.3942	0.0074	0.0023	0.0030
1	0.0426	0.0893	0.1033	0.0019	0.0006	0.0006
1	0.0788	0.1224	0.4748	0.0026	0.0010	0.0015
1	0.0527	0.1589	0.2260	0.0016	0.0012	0.0007
1	0.0322	0.2459	0.1560	0.0016	0.0004	0.0004
1	0.0452	0.0832	0.2903	0.0033	0.0009	0.0010
1	0.1833	0.5282	0.5859	0.0064	0.0027	0.0023
**TD**						
2	0.1191	0.0812	0.1415	0.0013	0.0022	0.0020
2	0.0657	0.1096	0.2497	0.0017	0.0022	0.0013
2	0.0998	0.0743	0.2858	0.0025	0.0009	0.0013
2	0.0965	0.1360	0.3082	0.0023	0.0016	0.0013
2	0.0231	0.1937	0.2289	0.0025	0.0010	0.0006
2	0.3144	0.3258	0.4231	0.0056	0.0021	0.0044
2	0.0376	0.1298	0.1110	0.0018	0.0003	0.0007
2	0.0359	0.1012	0.2624	0.0012	0.0010	0.0006
2	0.0381	0.1800	0.1977	0.0022	0.0023	0.0011
2	0.0281	0.0762	0.1060	0.0015	0.0008	0.0006
2	0.0433	0.0745	0.1944	0.0018	0.0006	0.0009
2	0.0969	0.1548	0.2143	0.0015	0.0023	0.0011
2	0.0566	0.0831	0.1272	0.0018	0.0006	0.0010
2	0.0564	0.1403	0.2922	0.0018	0.0022	0.0014
2	0.0828	0.2080	0.1962	0.0016	0.0012	0.0011
